# Abacavir forms novel cross-linking abacavir protein adducts in patients

**DOI:** 10.1186/2045-7022-4-S3-P38

**Published:** 2014-07-18

**Authors:** Xiaoli Meng, Alexandre Lawrenson, Neil Berry, James Maggs, Neil French, David Back, Saye Khoo, Dean Naisbitt, Kevin Park

**Affiliations:** 1 Liverpool University, MRC Centre for Drug Safety Science, UK

## Background

Abacavir (ABC), a nucleoside-analogue reverse transcriptase inhibitor, is associated with severe hypersensitivity reactions that involve the activation of CD8+ T cells in a HLA-B*57:01-restricted manner. Recent studies have claimed that non-covalent interactions of ABC with HLA-B*57:01 are responsible for the immunological reactions associated with ABC. However, the formation of haemoglobin-ABC aldehyde (ABCA) adducts in patients exposed to ABC suggests that protein conjugation might represent a pathway for antigen formation.

## Method

To further characterize protein conjugation reactions, we used mass spectrometric methods to define ABCA modifications of human serum albumin and glutathione S-transferase Pi *in vitro* and in patients commencing ABC therapy.

## Results

ABCA formed a novel intramolecular cross-linking adduct on human serum albumin in patients and *in vitro* via Michael addition, followed by nucleophilic adduction of the aldehyde with a neighbouring protein nucleophile. Adducts were detected on lysine, histidine, and cysteine residues in subdomain IB of human serum albumin. Only a cysteine adduct and a putative cross-linking adduct were detected on glutathione S-transferase Pi. Modelling the docking of ABCA with HLA B*57:01 confirmed that ABCA has a strong binding affinity when bound covalently to Ser116, a key residue with regards to recognition by ABC-specific CD8+ T cells.

## Conclusion

These findings reveal that ABC forms novel types of haptens in all patients taking the drug. It is therefore vital that the immunological consequences of such haptenation pathways are explored in the *in vitro* models that have been used by various groups to define a new mechanism of drug hypersensitivity for ABC.

